# Linking Dual-Pathway Carbon Deoxidation Kinetics to Melt-Level Rise and Splash Risk in Molten Steel

**DOI:** 10.3390/ma19173754

**Published:** 2026-09-03

**Authors:** Xiwen Zhang, Chao Gu, Fang Gao, Yanping Bao

**Affiliations:** 1 State Key Laboratory of Advanced Metallurgy, University of Science and Technology Beijing, Beijing 100083, China; m202421586@xs.ustb.edu.cn; 2 China Petroleum Materials Procurement Center, China National Petroleum Corporation, Beijing 100029, China; 18331563709@163.com

**Keywords:** carbon deoxidation, splash prevention, dual-pathway kinetic model

## Abstract

Carbon deoxidation is a clean steelmaking route, but rapid CO generation can increase gas holdup, raise the melt level, and cause splashing. However, a quantitative link between internal deoxidation kinetics and melt-level rise has not yet been established. Hot-state experiments at 1873 K in a 2 kg crucible under argon with 0.20–1.00 wt.% C were coupled with a dual-pathway kinetic model developed by decoupling free-surface mass transfer from internal CO generation through heterogeneous nucleation at active refractory pores and incorporating an effective reaction depth constrained by hydrostatic pressure. The model reproduced measured oxygen evolution and revealed three deoxidation stages. Internal gas generation initially dominated, contributing 96.6% of the overall rate. As carbon–oxygen supersaturation fell below a critical threshold, the nucleation zone receded upward and free-surface mass transfer became dominant. Within the investigated conditions, the internal apparent deoxidation rate constant during the rapid stage showed an empirical linear relationship with the measured maximum melt-level rise. The critical splash-prevention rate was 4.25 min^−1^ for the crucible and was estimated to be 2.43 min^−1^ for a selected 85 t ladle by combining this relationship with the average gas holdup relation and ladle geometry. The present results provide a kinetic basis for assessing splash risk during atmospheric carbon deoxidation.

## 1. Introduction

High performance and long service life remain primary objectives in steel materials development, placing ever-stricter demands on molten steel cleanliness [1,2,3]. Ladle refining constitutes a key stage for improving cleanliness, with deoxidation directly governing the total oxygen content [4,5]. Oxide inclusions generated by traditional metallic deoxidizers, such as aluminum and silicon, however, are difficult to eliminate completely. They frequently cause submerged entry nozzle clogging during continuous casting and impair the fatigue resistance of final products [6,7,8,9]. Carbon deoxidation offers an alternative clean process because its product, CO gas, readily escapes the melt without leaving solid inclusions [10,11]. The rapid carbon–oxygen reaction at atmospheric pressure nevertheless accelerates internal CO gas generation. Abundant bubbles trapped in the melt raise gas holdup, directly driving macroscopic melt-level rise and splashing [12]. The coupling between microscopic deoxidation kinetics and macroscopic fluid behavior must therefore be clarified before atmospheric carbon deoxidation can be implemented industrially.

Melt deoxidation spans cross-scale phenomena from microscopic bubble evolution to macroscopic melt expansion. Gu et al. [13] established a mathematical model coupling liquid boundary layer mass transfer with dynamic bubble growth, validating bubble expansion behavior through in situ gas generation from the thermal decomposition of an ammonium bicarbonate solution. Their results indicate that initial oxygen content serves as the primary driving force for bubble growth. At an initial oxygen content of 500 ppm, the interfacial reaction drove a single CO bubble to expand by a factor of 12.39, markedly accelerating bubble ascent and inducing melt volume expansion. In a vacuum RH degasser, Takahashi et al. [14] spatially decoupled the reaction interfaces into three independent zones: argon bubbles, the free surface, and internal CO bubbles. Comparison between their calculations and industrial data confirmed that thermodynamically driven internal CO bubbles contributed the vast majority of decarburization during refining, with subsequent rate decay directly attributable to the depletion of internal bubble generation. Geng et al. [15] subsequently adopted this framework in numerical simulations to investigate the effects of ladle bottom blowing on decarburization and inclusion removal in the RH process. Their simulations revealed that bottom-blown bubbles enhanced bath circulation and expanded the internal CO bubble generation zone, allowing the internal deoxidation pathway to dominate throughout the early refining stage.

Guo and Irons [16] constructed a three-dimensional gas–liquid two-phase model that spatially quantified interfacial mass transfer by allocating it to both the surfaces of ascending bubbles and the melt-free surface, providing a 3D numerical foundation for the regional decoupling of deoxidation pathways. Beyond governing the metallurgical reaction, massive internal CO bubble generation directly raises gas holdup and triggers macroscopic melt-level rise. Cicutti et al. [17] measured through incremental sampling in a 200-ton industrial converter and found that during the peak decarburization period, the apparent gas velocity induced by internal reactions could reach 3.5 m·s^−1^. This intense gas generation significantly elevated the gas holdup and foaming index, pushing the system close to the physical threshold for splashing. Wang et al. [18] employed physical modeling to determine the quantitative relationship between gas velocity and melt behavior. Their results revealed that within a specific range of apparent gas velocity, the foam rise height exhibited a strictly linear correlation with the apparent gas velocity. Once the liquid level exceeded the vessel’s freeboard, macroscopic splashing occurred.

Despite these advancements in bubble evolution and carbon deoxidation thermodynamics, theoretical limitations persist for atmospheric ladle refining systems. Existing spatially decoupled models predominantly focus on vacuum RH decarburization or bottom-blown gas–liquid mass transfer, with objectives largely limited to quantifying the total removal rate. Although some studies have addressed the effect of hydrostatic pressure on internal CO bubble generation, a quantitative correlation between the microscopic kinetics of internal deoxidation and macroscopic melt-level rise remains elusive. Industrial operations therefore lack reliable kinetic indicators for predicting the extent of melt expansion, making quantitative operating boundaries for splash prevention difficult to establish.

Accordingly, this study aims to establish a quantitative link between internal carbon-deoxidation kinetics and melt-level rise under atmospheric refining conditions. Hot-state experiments were conducted at 1873 K in a 2 kg crucible under an argon atmosphere, with initial carbon contents of 0.20, 0.45, and 1.00 wt.%, and were complemented by numerical modeling. A dual-pathway kinetic model was developed to distinguish deoxidation at the free surface from internal CO generation at active refractory pores while accounting for an effective reaction depth constrained by hydrostatic pressure. The model was used to characterize the temporal evolution of the two deoxidation pathways. During the initial rapid deoxidation stage, the internal apparent deoxidation rate constant was correlated with the measured maximum melt-level rise. This correlation was subsequently used to determine the critical rate for preventing splashing in the crucible. The industrial estimate was obtained by combining this relationship with a scaling approach based on average gas holdup and the geometry of an 85 t ladle. The corresponding allowable dissolved oxygen content at ladle entry was then evaluated to provide a control target for the converter deoxidation endpoint. The resulting framework establishes a quantitative link between microscopic CO-generation kinetics and macroscopic melt expansion and provides a basis for assessing splash risk during atmospheric carbon deoxidation.

## 2. Experimental

### 2.1. Experimental Method

Hot-state carbon deoxidation experiments were conducted in a 2 kg VIF-2 vacuum induction furnace. Reactions took place within an induction-heated magnesia crucible that simulates atmospheric ladle refining using a small-scale melt pool. The crucible had a uniform cylindrical cross-section, and the melt-free surface was maintained under argon at atmospheric pressure. Prior to melting the charge, the furnace chamber was evacuated to below 10 Pa using a vacuum pump and subsequently backfilled with high-purity argon to 101,325 Pa, equivalent to 1 atm. This evacuation and backfilling cycle was repeated three times to ensure an inert environment. The entire deoxidation reaction was therefore conducted under atmospheric argon protection. Table 1 details the geometric dimensions of the crucible, including the critical freeboard *H*_f_, and the physical properties of the melt.

Raw materials employed in the experiments comprised high-purity electrolytic iron, 99.99% pure Fe_2_O_3_ powder, and a bituminous coal recarburizer. The electrolytic iron served as the steel matrix, while the Fe_2_O_3_ powder was introduced to establish the initial dissolved oxygen content (*w*_[O]0_) prerequisite for the reaction. The bituminous coal recarburizer was specifically utilized to set the initial carbon content (*w*_[C]0_) of the melt. The proximate analysis of the pulverized coal additive is presented in Table 2.

### 2.2. Experimental Procedure and Composition Analysis

**Figure 1:**
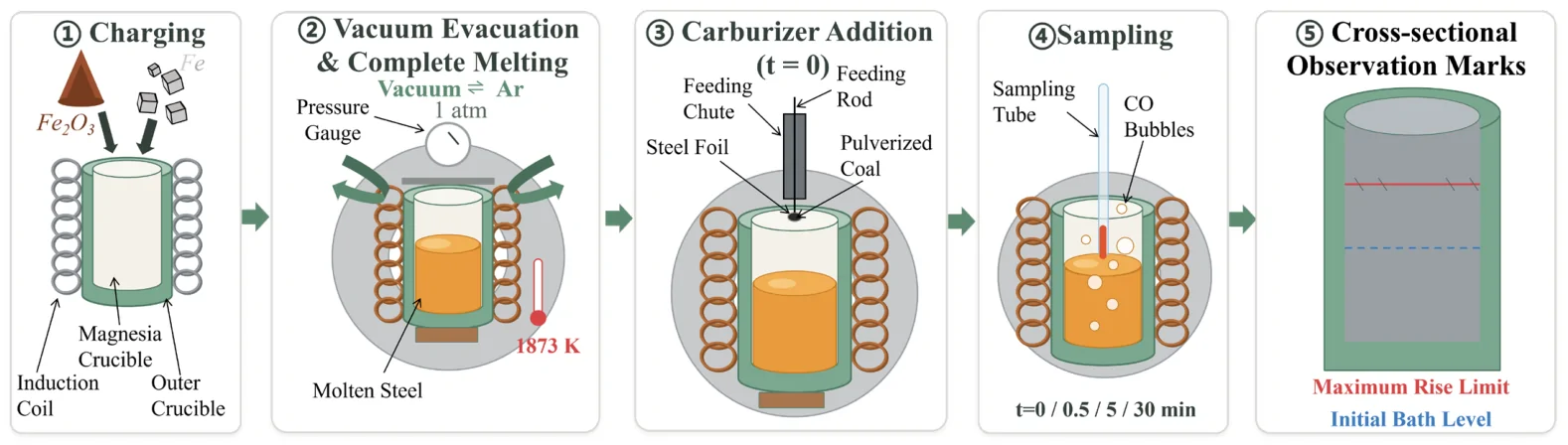
Schematic of the carbon deoxidation hot-state experimental procedure.

The process began with loading electrolytic iron and Fe_2_O_3_ powder into the magnesia crucible, with no slag layer present during the experiment, as shown in the experimental workflow in Figure 1. Following atmosphere replacement, the charge was heated until completely molten. After the bath surface became quiescent and the temperature stabilized at 1873 K, the bituminous coal recarburizer was wrapped in steel foil with a thickness of 0.01 mm, secured to the feeding rod with iron wire, and immersed in the molten steel. This submerged addition procedure minimized premature surface reactions and melt eruption during carbon addition [19]. The exact moment of recarburizer addition was defined as the reaction zero point, *t* = 0. Melt samples were taken at 0, 0.5, 5, and 30 min to capture the kinetic evolution. After 30 min, heating was terminated and the system was allowed to cool.

The initial carbon contents were established at 0.20 wt.%, 0.45 wt.%, and 1.00 wt.% to represent low-, medium-, and high-carbon systems, respectively, and to examine the applicability of the proposed framework over this carbon-content range. The initial oxygen contents measured using a Leco TCH-600 oxygen/nitrogen analyzer (TCH-600, LECO Corporation, St. Joseph, MI, USA) were 404, 332, and 370 ppm for the melts containing 0.20, 0.45, and 1.00 wt.% C, respectively. The target initial carbon contents were calculated from the recarburizer addition masses. The actual carbon contents used as model inputs, together with the carbon contents at the subsequent sampling times and the remaining elemental compositions, were measured using a direct-reading spectrometer (ARL-8860, Thermo Fisher Scientific, Waltham, MA, USA).

## 3. Modeling

### 3.1. Thermodynamic and Nucleation Conditions of Carbon Deoxidation

Under atmospheric steelmaking conditions at 1873 K, the carbon deoxidation reaction is governed by Equation (1):

(1) [C] + [O] = CO(g)

The thermodynamic equilibrium constant *K* is determined by Equation (2):

(2) $$\mathrm{lg}K\;=\;\frac{1168}{T}\;+\;2.07$$

where *T* is the thermodynamic temperature, set to 1873 K. This equilibrium relationship gives the theoretical equilibrium partial pressure of CO, denoted as *P*_CO_, through Equation (3):

(3) $$P_{\mathrm{CO}}\;=\;Kw_{\left[C\right]}w_{\left[O\right]}$$

where *w*_[C]_ and *w*_[O]_ represent the mass fractions of carbon and oxygen in the molten steel in wt.%.

The product of the carbon and oxygen activity coefficients is taken as unity in the composition range studied [11]. This approximation permits Equation (3) to be written with the measured mass fractions. According to homogeneous nucleation theory, a CO bubble must exceed a critical nucleus radius to form and grow stably within the molten steel. A forming CO bubble must overcome atmospheric pressure, the hydrostatic pressure of the melt, and the Laplace pressure arising from surface tension. This mechanical condition is expressed by Equation (4):

(4) $$P_{g}\;\geq P_{0}\;+\;\rho gh\;+\;\frac{2\sigma }{r_{c}}$$

where *P*g is the CO gas pressure within the bubble in Pa; *P*_0_ is the ambient pressure of 101,325 Pa; *ρ* is the density of the molten steel at 7200 kg·m^−3^; g is the gravitational acceleration at 9.81 m·s^−2^; *h* is the nucleation depth from the melt-free surface in m; *σ* is the surface tension of the molten steel in N·m^−1^; and *r*_c_ is the curvature radius of the bubble in m.

The Laplace pressure exerted on a micro-bubble nucleus is inversely proportional to its radius, and a smaller initial bubble size necessitates higher supersaturation to sustain the nucleus [20]. Maintaining a stable CO bubble nucleus within the actual melt requires the instantaneous localized aggregation of 10^7^ to 10^11^ CO molecules. Such massive molecular clustering is extremely difficult to achieve solely through localized concentration fluctuations within a macroscopic homogeneous melt. Homogeneous nucleation is therefore virtually impossible within the bulk molten steel [21,22].

The critical radius required for stable CO bubble formation decreases with increasing supersaturation [20]. In contrast, the surface of refractory material contacting the molten steel contains numerous gas-filled micropores. Due to the poor wettability of molten steel on the refractory, these pores retain pre-existing gas–liquid interfaces that can serve as heterogeneous nucleation sites for the carbon–oxygen reaction, effectively lowering the surface tension barrier [23]. However, only some pores can act as effective nucleation sites that satisfy the mechanical equilibrium condition defined by Equation (5):

(5) $$P_{g}\;\geq \;P_{0}+\frac{2\sigma \cos\theta }{R}+\rho gh$$

where *θ* is the contact angle between the molten steel and the refractory pores, taken as 150°, and *R* is the refractory pore radius in m. Two further constraints determine the allowable range of active pore sizes. If the pore radius exceeds an upper limit, the molten steel penetrates the pore under hydrostatic pressure, thereby disabling it as a bubble nucleus. Equation (6) bounds this condition:

(6) $$R_{\max}=-\frac{2\sigma \cos\theta }{\rho gh}$$

Conversely, for a bubble to grow sufficiently and detach from the pore orifice, it must overcome the Laplace pressure. When the pore radius falls below the minimum threshold *R*_min_, the bubble is hindered by surface tension and cannot detach. Equation (7) mathematically constrains this condition:

(7) $$R_{\min}=\frac{2\sigma \cos\theta }{P_{g}{\;-\;P}_{0}\;-\;\rho gh}$$

A pore is therefore defined as an active pore only when its radius falls strictly between these two limits.

The macroscopic thermodynamic prerequisite for continuous bubble generation from pores is that the internal phase-transformation pressure exceeds the total external pressure. As the local depth of the molten steel increases to the point where *P*_g_ equals *P*_0_ plus *ρ*g*h*, the thermodynamic driving force for bubble generation is completely exhausted at this depth, irrespective of pore dimensions, causing all active pores to become inactive. This critical depth defines the effective reaction depth *H*, within which internal deoxidation is thermodynamically feasible. Equation (8) formulates this depth:

(8) $$H\;=\;\frac{P_{g}\;-\;P_{0}}{\rho g}$$

The entire melt pool is therefore spatially partitioned by the depth *H* into two distinct regions: the upper heterogeneous nucleation zone driven by supersaturation, and the deeper inactive zone suppressed by hydrostatic pressure. To quantify the driving force of the deoxidation reaction, this study introduces a dimensionless carbon–oxygen supersaturation, *Π*. Equation (9) defines it as the ratio of the theoretical equilibrium partial pressure of CO bubbles to the standard atmospheric pressure *P*^0^:

(9) $$\Pi \;=\;\frac{P_{\mathrm{CO}}}{P^{0}}$$

where *P*^0^ is the standard atmospheric pressure of 1 atm used to normalize *P*_CO_. *Π* is a dimensionless, composition-based supersaturation index independent of depth.

### 3.2. Dual-Pathway Deoxidation Kinetic Model

**Figure 2:**
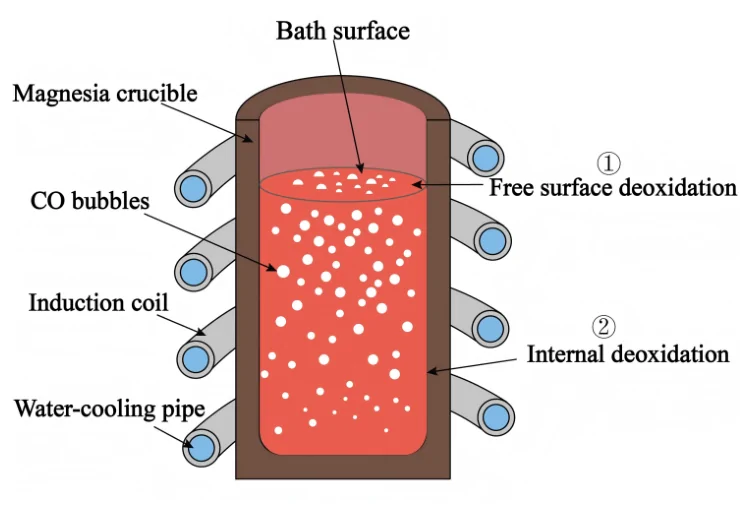
Schematic diagram of the reaction zone inside the crucible.

The above considerations indicate that the carbon deoxidation reaction within the atmospheric crucible predominantly occurs in three regions: the melt-free surface, splashing droplets, and the active refractory pores within the molten steel. Since splashing droplets promote the reaction by expanding the gas–liquid interface, their contribution is lumped into the free-surface deoxidation pathway to simplify the kinetic structure. The macroscopic deoxidation process is therefore spatially decoupled into two parallel primary pathways: free-surface deoxidation and internal pore deoxidation, as schematically illustrated in Figure 2.

#### 3.2.1. Free-Surface Deoxidation Kinetic Model

Liquid boundary-layer mass transfer governs the transport of dissolved oxygen to the free surface [24]. The classical two-film mass transfer theory gives the free-surface deoxidation rate *D*_[O]s_ through Equation (10):

(10) $$D_{[O]s}\;={\;k}_{s}\frac{A_{s}}{V}(w_{\left[O\right]}\;-\;w_{[O]e})$$

where *k*_s_ is the mass transfer coefficient of oxygen at the gas–liquid interface with a value of 1.644 × 10^−4^ m·s^−1^, and *A*_s_ and *V* are the free-surface area and the volume of the molten steel, respectively.

Under the argon atmosphere used in the experiments, the gas phase boundary condition is represented by a fixed equivalent CO partial pressure *P*_CO,furnace_. The effect of crucible-melt interactions is represented by an additional equivalent oxygen contribution. Accordingly, the thermodynamic CO equilibrium oxygen content and the effective interfacial oxygen content are defined separately by Equations (11) and (12):

(11) $$w_{[O],\mathrm{CO}}^{\mathrm{eq}}\;=\;\frac{P_{\mathrm{CO},\mathrm{furnace}}}{Kw_{\left[C\right]}}$$

(12) $$w_{[O]e}\;={\;O}_{\mathrm{cru}\;}+\;\frac{P_{\mathrm{CO},\mathrm{furnace}}}{Kw_{\left[C\right]}}$$

where *P*_CO,furnace_ is the CO partial pressure in the furnace, fixed at 0.044 atm; O_cru_ is the fitted equivalent oxygen contribution associated with interactions between the crucible and the melt; $w_{[O],CO}^{eq}$ is the CO equilibrium oxygen content calculated by Equation (11), and *w*_[O]e_ is the effective interfacial oxygen content calculated by Equation (12).

#### 3.2.2. Internal Deoxidation Kinetic Model

Because active pores are depth-dependent and the detaching CO bubbles continuously serve as deoxidation interfaces during their ascent, this hydrostatically suppressed thermodynamic process must be quantitatively resolved. Following the reaction zone model proposed by Kuwabara et al. [14,25,26] for internal deoxidation in an RH vacuum vessel, this process is treated using a depth integral that accounts for the hydrostatic pressure gradient and thermodynamic supersaturation. A depth-integrated deoxidation kinetic model is therefore established. Equation (13) gives the internal deoxidation rate *D*_[O]i_:

(13) $$D_{[O]i}\;=\;k_{a}\frac{A_{s}}{V}\int_{0}^{H}(w_{\left[O\right]}\;-\;w_{{\left[O\right]}_{h}^{\mathrm{eq}}})\;dh$$

(14) $$w_{{\left[O\right]}_{h}^{\mathrm{eq}}}\;=\;\frac{P_{0}\;+\;\rho gh}{P_{0}Kw_{\left[C\right]}}$$

where *k*_a_ is the process coefficient for the internal deoxidation pathway, and $w_{{\left[O\right]}_{h}^{eq}}$ is the static-pressure equilibrium oxygen content at the corresponding depth.

### 3.3. Numerical Solution of the Kinetic Model

Quantitative prediction of the dynamic evolution of the dual-pathway deoxidation process was achieved by constructing a system of governing ordinary differential equations for solute consumption based on stoichiometric relationships, as expressed by Equations (15) and (16):

(15) $$\frac{dw_{\left[O\right]}}{dt}\;=\;-(D_{[O]s}\;+{\;D}_{[O]i})$$

(16) $$\frac{dw_{\left[C\right]}}{dt}\;=\;0.75\frac{dw_{\left[O\right]}}{dt}$$

**Figure 3:**
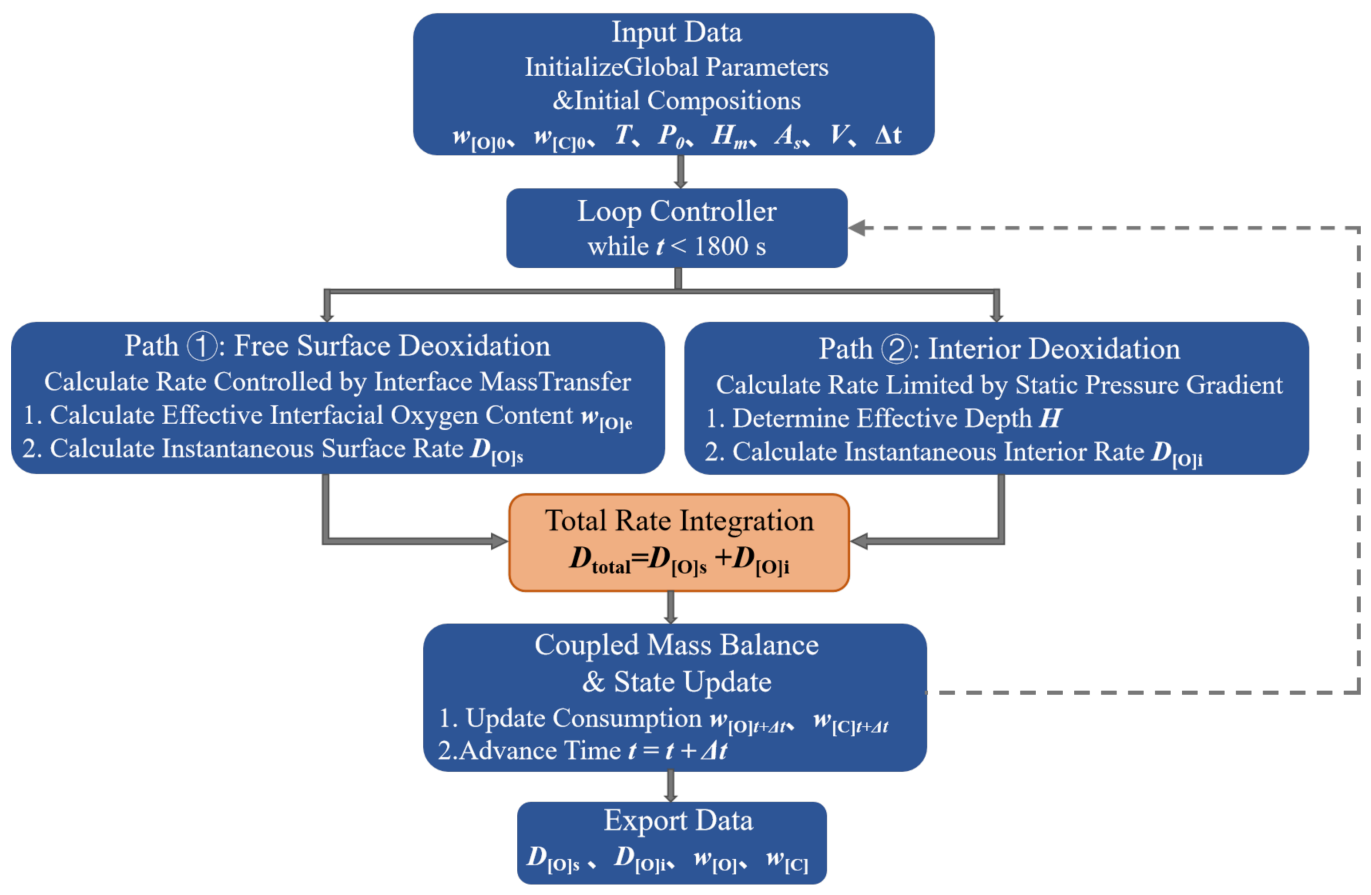
Numerical logic diagram of the dual-pathway deoxidation model.

In the model, the dissolved carbon and oxygen contents were assumed to be spatially uniform throughout the melt. This assumption is consistent with the strong mixing caused by induction stirring [27,28]. This assumption applies to the dissolved solutes in the molten steel and does not describe the dissolution process of individual recarburizer particles. Given the initial carbon content *w*_[C]0_ and initial dissolved oxygen content *w*_[O]0_, an explicit time-stepping method was employed to numerically solve the model. The iteration step size Δ*t* was set to 0.1 s, covering a total computational duration of 1800 s. Within each time step, the model dynamically evaluates the carbon–oxygen supersaturation *Π* and the effective reaction depth *H* based on the transient melt composition. The free-surface deoxidation rate *D*_[O]s_ and the internal deoxidation rate *D*_[O]i_ are then independently calculated to update the solute concentrations for the subsequent time step. This iteration continues until the reaction is complete. Figure 3 shows the numerical logic of this computational procedure.

The model calculates the reaction kinetics, solute consumption, and effective reaction depth. It does not solve the gas–liquid flow field or the motion of individual bubbles. The melt-level response is introduced through the relationship established based on the experimental results in Section 4.3.

All variables and parameters used in the kinetic model, together with their units, definitions, and values, are summarized in Appendix A.

## 4. Results and Discussion

### 4.1. Parameter Fitting and Model Validation

The reliability of the proposed dual-pathway carbon deoxidation kinetic model under atmospheric crucible conditions was assessed using the measured oxygen evolution. The process coefficient k_a_ was calibrated by trial and error. During the calibration, k_a_ was varied and the calculated oxygen evolution was compared with the hot-state experimental data. A value of k_a_ = 0.08 s^−1^ provided satisfactory agreement and was therefore adopted in the model. The equivalent crucible oxygen contribution O_cru_ was determined by fitting. The fitted parameters are also included in the consolidated parameter list provided in Appendix A.

**Figure 4:**
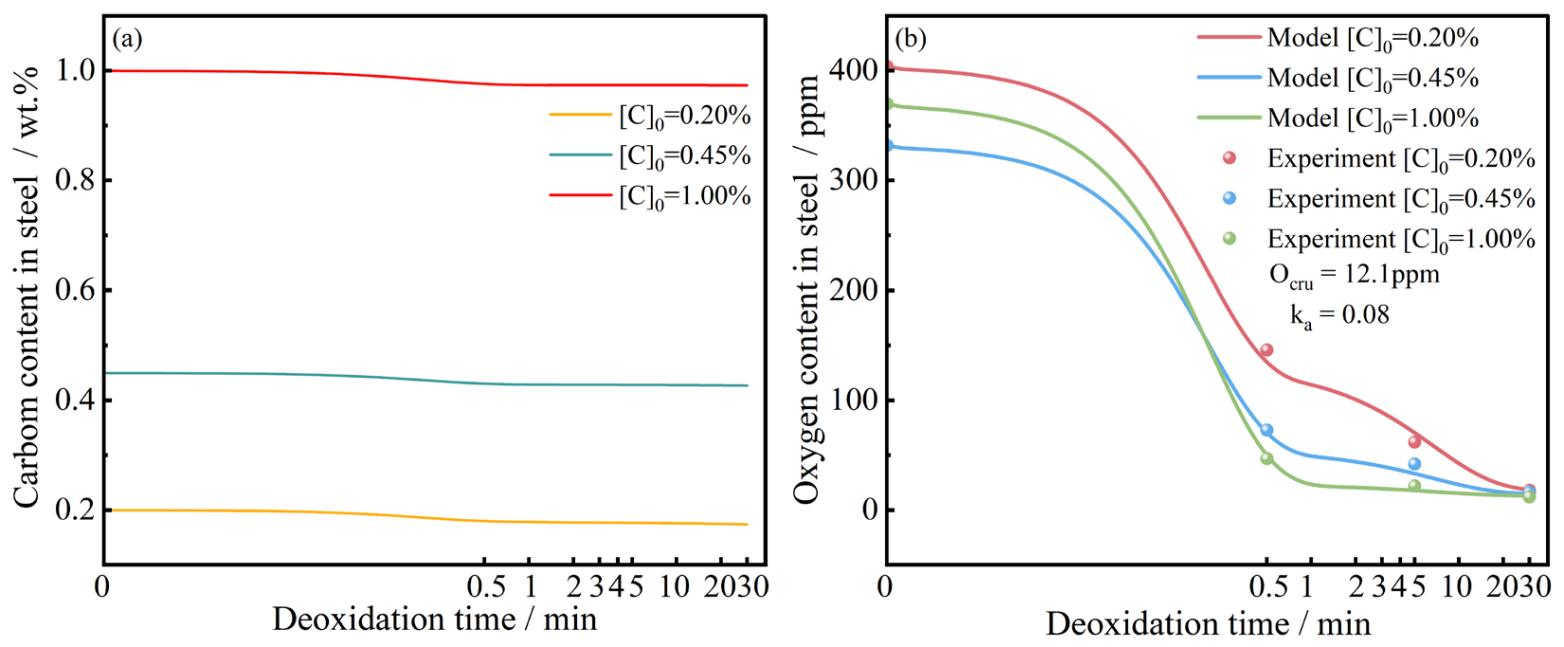
Temporal evolution of melt composition under different initial carbon contents: (**a**) calculated carbon content; (**b**) calculated and measured oxygen content.

The temporal evolution of carbon content was calculated according to the carbon–oxygen reaction stoichiometry. Figure 4a presents the calculated carbon-content curves, whereas Figure 4b compares the calculated and measured oxygen contents under the three initial carbon conditions. The solid curves in Figure 4b denote the model predictions, and the symbols represent the experimental measurements at 0, 0.5, 5, and 30 min. The figure also provides the calibrated value of k_a_ and the fitted value of O_cru_.

As shown in Figure 4a, carbon is consumed predominantly during the initial rapid-deoxidation stage and subsequently approaches a nearly stable value as dissolved oxygen and carbon–oxygen supersaturation decrease. Based on the stoichiometric relationship between carbon and oxygen consumption, the calculated carbon contents after 30 min are approximately 0.171, 0.426, and 0.973 wt.% for initial carbon contents of 0.20, 0.45, and 1.00 wt.%, respectively. The relative carbon-content change remains limited because dissolved oxygen is the limiting reactant, but its temporal variation is fully incorporated into the mass-balance calculation rather than being treated as constant.

As shown in Figure 4b, within the first 0.5 min the system undergoes a rapid deoxidation stage during which the oxygen content under all three experimental conditions decreases sharply and nonlinearly. For the system with the highest initial carbon content of 1.00 wt.%, the measured oxygen content decreases rapidly from 370 ppm to 47 ppm, corresponding to the removal of approximately 87% of the dissolved oxygen within this period. Subsequently, the reaction rate decreases significantly. After 30 min of reaction, the system approaches a dynamic equilibrium state, and the measured residual oxygen contents for the three conditions reach 18, 16, and 12 ppm, respectively. The calculated *w*_[O]_ curves reproduce the initial nonlinear decrease well, and the predicted oxygen contents at 30 min of 18.6, 14.7, and 13.2 ppm show good agreement with the experimental measurements.

The sum of squared relative errors between predicted and measured values is 0.126, with a mean relative error of 7.7%. This agreement suggests that, over the carbon content range of 0.20 wt.% to 1.00 wt.%, the proposed dual-pathway model reliably describes the dynamic evolution of dissolved oxygen concentration during the deoxidation process.

To assess the robustness of the adopted parameter set, a local sensitivity analysis was performed by independently varying *k*_s_, *k*_a_, and O_cru_ by ±10% while holding all other parameters and initial conditions fixed, without subsequent parameter refitting. The corresponding SSE ranges were 0.1317–0.1325 for *k*_s_, 0.1440–0.1718 for k_a_, and 0.1458–0.1488 for O_cru_. The normalized local sensitivity matrix gave a maximum absolute parameter correlation of 0.44, indicating no strong local compensation among the three parameters.

### 4.2. Evolution of Deoxidation Kinetics

**Figure 5:**
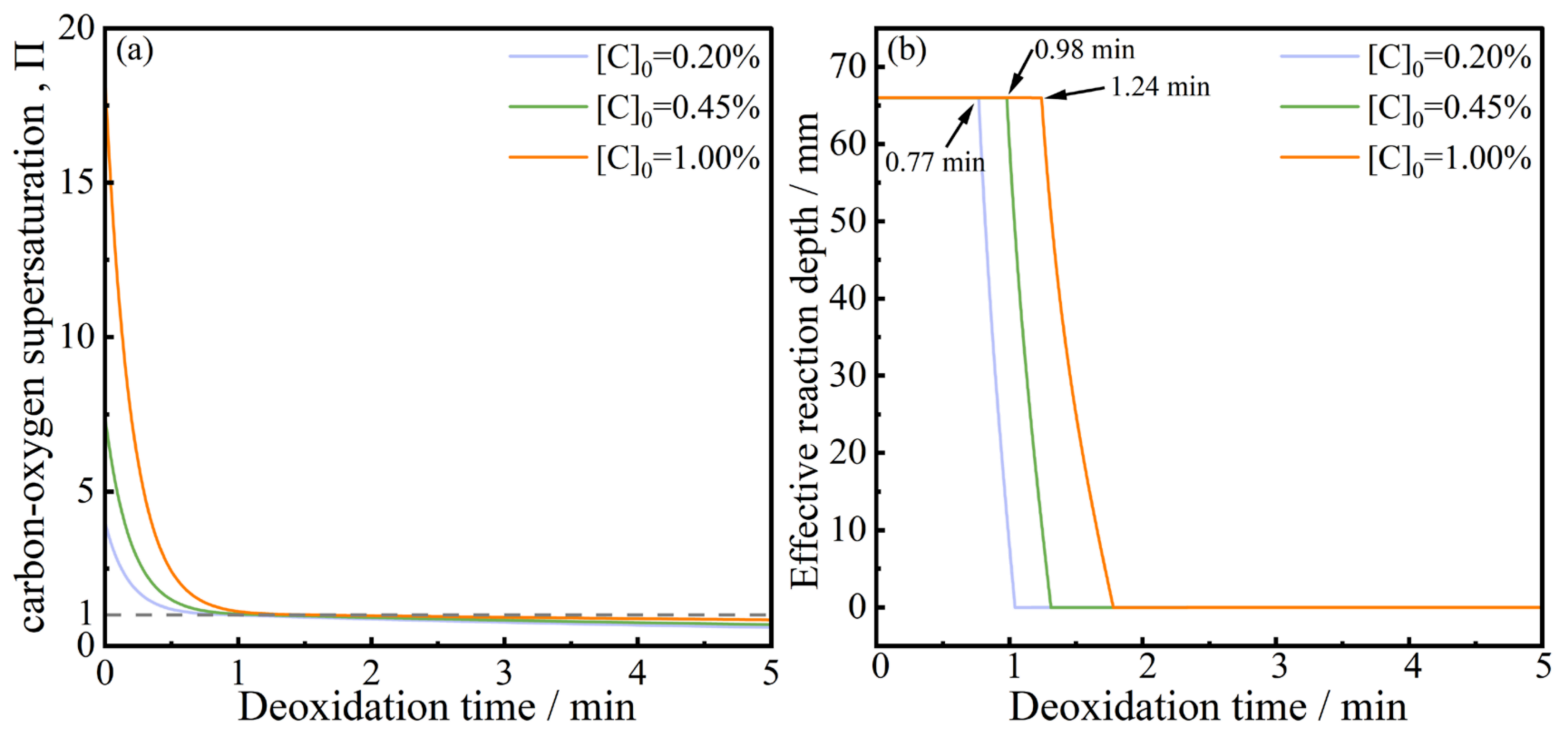
(**a**) Carbon–oxygen supersaturation; (**b**) effective reaction depth.

Deoxidation proceeds through three distinct stages at initial carbon contents of 0.20, 0.45, and 1.00 wt.%. Throughout the process, deoxidation is governed primarily by the gradual decay of carbon–oxygen supersaturation. As supersaturation decreases, the thermodynamic driving force for bubble nucleation weakens, causing the effective reaction depth to retreat upward from the pool bottom. Consequently, contraction of the reaction volume sharply reduces the internal deoxidation contribution fraction, shifting the dominant deoxidation pathway. Figure 5 presents the corresponding evolution of supersaturation and effective reaction depth.

During stage S1, corresponding to the first 0.5 min, high local supersaturation provides sufficient thermodynamic driving force for internal CO nucleation. The initial supersaturation *Π*_0_ increases markedly with increasing carbon content, reaching 3.99, 7.38, and 18.27 for the low-, medium-, and high-carbon systems, respectively, as shown in Figure 5a.

When the local supersaturation exceeds the combined ambient and hydrostatic pressures at the pool bottom, the reaction front extends throughout the melt pool. All three systems therefore maintain the maximum depth of 0.066 m, as indicated in Figure 5b.

**Figure 6:**
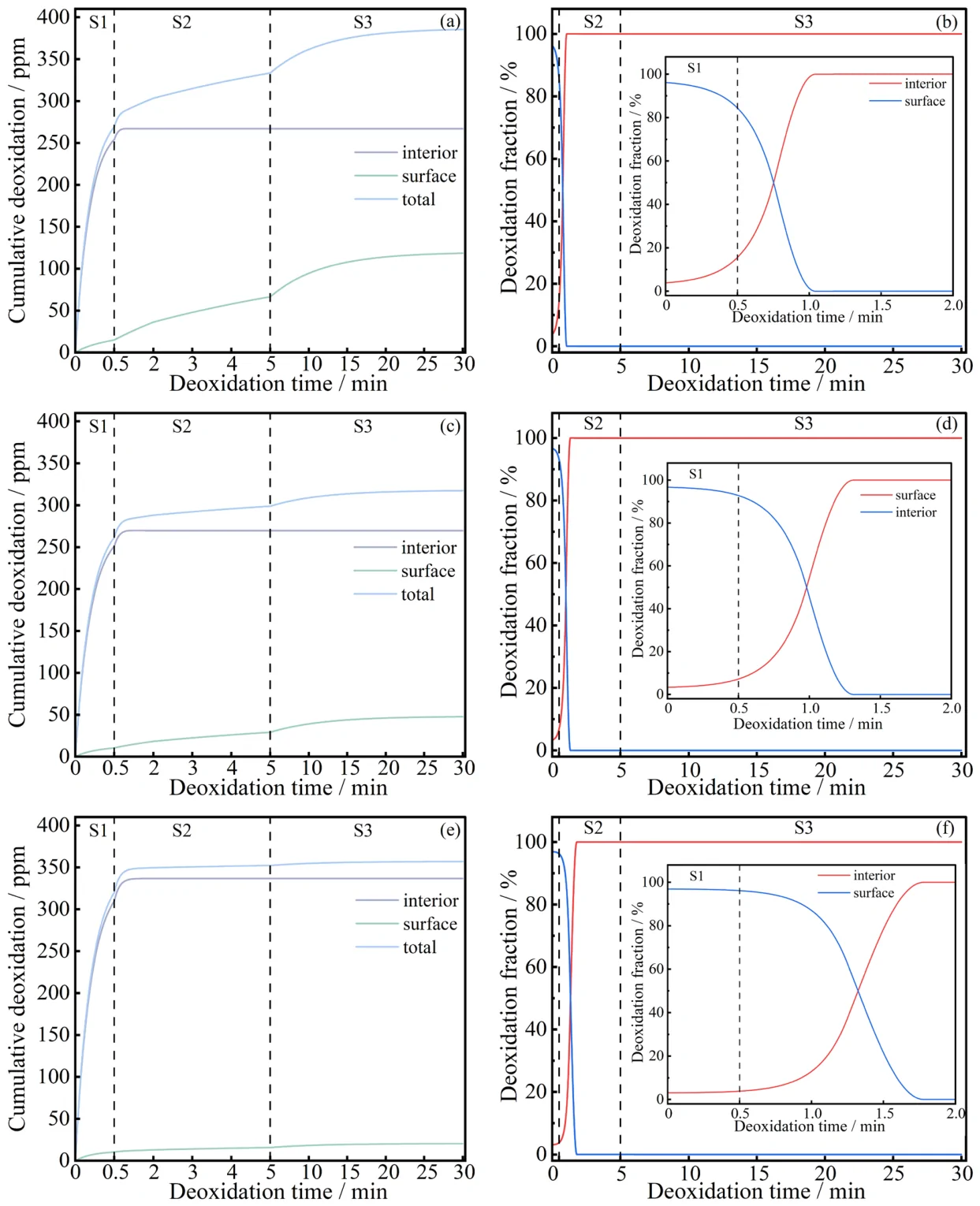
Evolution of deoxidation paths under different initial carbon contents: (**a**) cumulative deoxidation with [C]_0_ = 0.20%; (**b**) deoxidation fraction with [C]_0_ = 0.20%; (**c**) cumulative deoxidation with [C]_0_ = 0.45%; (**d**) deoxidation fraction with [C]_0_ = 0.45%; (**e**) cumulative deoxidation with [C]_0_ = 1.00%; (**f**) deoxidation fraction with [C]_0_ = 1.00%.

At the beginning of deoxidation, internal kinetic contribution fractions reach 94.1%, 96.6%, and 98.2% for the three systems, as shown in Figure 6b,d,f. Rapid CO generation quickly consumes dissolved oxygen, with oxygen contents dropping to 148, 71, and 47 ppm by 0.5 min, respectively. Residual supersaturation remains sufficient to sustain nucleation throughout the melt depth, so the effective reaction depth remains unchanged and the internal deoxidation contribution fraction stays above 89.5%.

As dissolved oxygen continues to be consumed, the dominant mass-transfer pathway switches during the 0.5–5 min interval, which is designated as stage S2. At the pool bottom, the chemical reaction driving force gradually balances with the hydrostatic pressure gradient, and the nucleation zone begins to shrink upward, reducing the effective reaction depth below 0.066 m. The model-predicted critical inflection points for the low-, medium-, and high-carbon systems occur at 0.77, 0.98, and 1.24 min, respectively, as shown in Figure 5b. Beyond these critical points, the active pore zone migrates from the bottom toward the free surface, while both the internal effective reaction volume and the gas generation flux decrease simultaneously.

As oxygen content approaches the theoretical equilibrium value and supersaturation tends toward 1.00, the thermodynamic condition for internal nucleation is lost. The calculated effective reaction depths *H* of the three systems reach zero at 1.00, 1.30, and 1.74 min, respectively, while the internal deoxidation contribution fraction curves in Figure 6b,d,f decrease toward zero synchronously. During this transition, the dominant kinetic pathway shifts from internal gas generation to free-surface mass transfer.

By 5 min, internal deoxidation has ceased in all three systems, and the process is in stage S3. Internal gas generation becomes negligible, gas holdup decays to zero, and the melt surface gradually returns to a quiescent state. Residual deoxidation is governed by solute diffusion through the liquid boundary layer at the free surface, causing the oxygen content to approach the endpoint at a very low rate, as shown in Figure 6a,c,e. After 30 min, the endpoint oxygen contents of the three systems reach 18.6, 14.7, and 13.2 ppm, respectively, and the deoxidation reaction is essentially complete.

### 4.3. Relationship Between Internal Deoxidation Rate and Melt-Level Rise

Experimental observations show that increasing the deoxidation rate enhances CO generation. The resulting bubbles become trapped within the bulk melt, increasing gas holdup and producing a measurable rise in melt level. Excessive melt expansion allows the liquid surface to reach the crucible mouth, thereby triggering splashing and compromising both process safety and metallurgical stability. To establish a quantitative relationship between the apparent deoxidation rate constant and melt expansion, the maximum melt-level rise Δ*H* over the entire deoxidation process is adopted as the characteristic parameter. The crucible wall trace method was used to measure Δ*H*. When Δ*H* reaches 65 mm, the melt level coincides with the crucible mouth, representing the physical threshold for splashing. Accordingly, Δ*H* ≥ 65 mm is adopted as the splash criterion.

**Figure 7:**
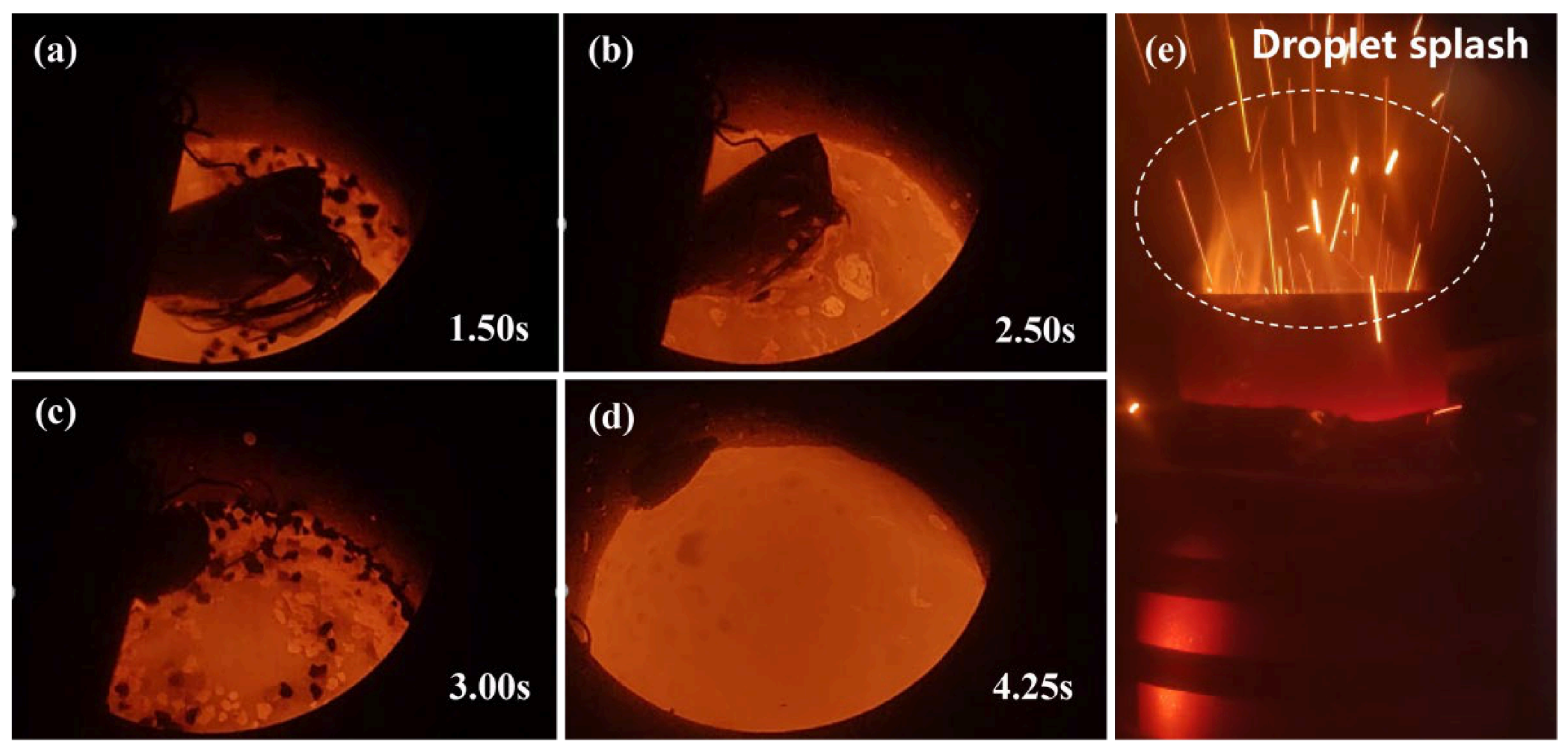
Rise in the bath surface and droplet splashing. (**a**) bath surface rise at 1.50 s; (**b**) bath surface rise at 2.50 s; (**c**) bath surface rise at 3.00 s; (**d**) bath surface rise at 4.25 s; (**e**) droplet splashing.

During stage S1, high local carbon–oxygen supersaturation provides sufficient driving force for internal deoxidation, making it the dominant pathway. CO bubbles generated within the melt increase gas holdup after being retained in the liquid, leading directly to melt-level rise and droplet splashing, as illustrated in Figure 7. In contrast, CO gas generated via free-surface deoxidation escapes directly into the gas phase and does not contribute to melt volume expansion. The internal deoxidation rate is described by the following equation:

(17) $$\;-\frac{dw_{{\left[O\right]}_{i}}}{dt}\;=\;K_{1}(w_{[O{]}_{i}}\;-\;w_{[O]e})$$

Integrating and linearizing yields:

(18) $$\;-\ln\frac{w_{{\left[O\right]}_{i,t}}\;-\;w_{[O]e}}{w_{{\left[O\right]}_{i,0}}\;-w_{[O]e}}\;=\;K_{1}\;t$$

where *K*_1_ is the internal apparent deoxidation rate constant (min^−1^).

**Figure 8:**
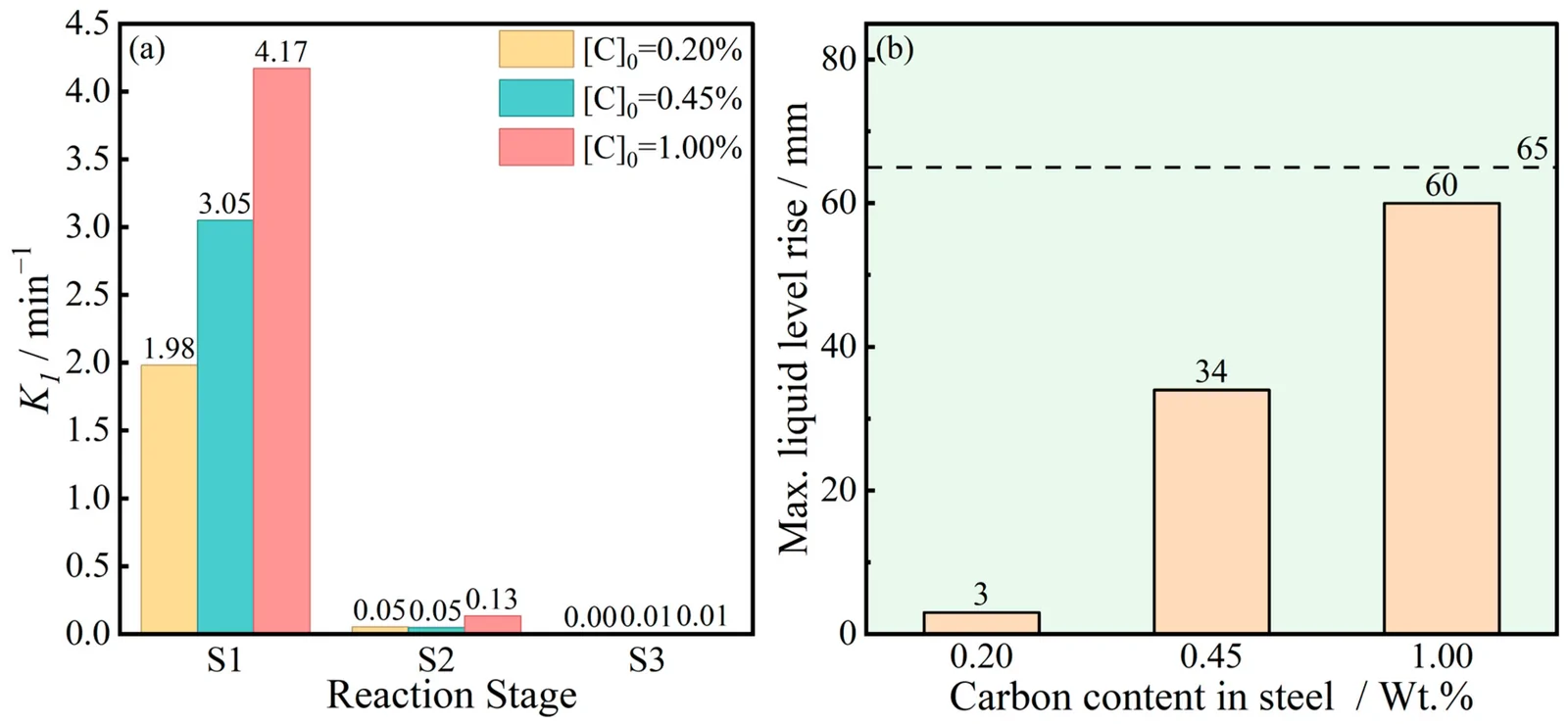
Melt-level rise and internal apparent deoxidation rate constant: (**a**) *K*_1_ at each stage; (**b**) maximum melt-level rise.

Figure 8 illustrates the relationship between the internal apparent deoxidation rate constant and melt-level rise under the three initial carbon–oxygen conditions. Most internal deoxidation occurs during stage S1, where *K*_1_ reflects the coupled influence of the initial carbon and oxygen contents. Across the three conditions with initial carbon contents increasing from 0.20% to 1.00%, *K*_1_ in stage S1 increases markedly to 1.98, 3.05, and 4.17 min^−1^, respectively. By contrast, deoxidation rates during stages S2 and S3 remain close to zero, indicating that most carbon–oxygen reactions are completed within a short period.

Correspondingly, differences in *K*_1_ are directly reflected in the observed melt-level rise, as illustrated in Figure 8b. A higher *K*_1_ promotes more rapid CO generation, resulting in a larger instantaneous bubble population. The accompanying increase in gas holdup produces a correspondingly larger rise in melt level. At an initial carbon content of only 0.20%, the melt-level rise is 3 mm, whereas at 0.45%, it reaches 34 mm, and at 1.00%, it reaches 60 mm, approaching the safety threshold under the operating conditions.

**Figure 9:**
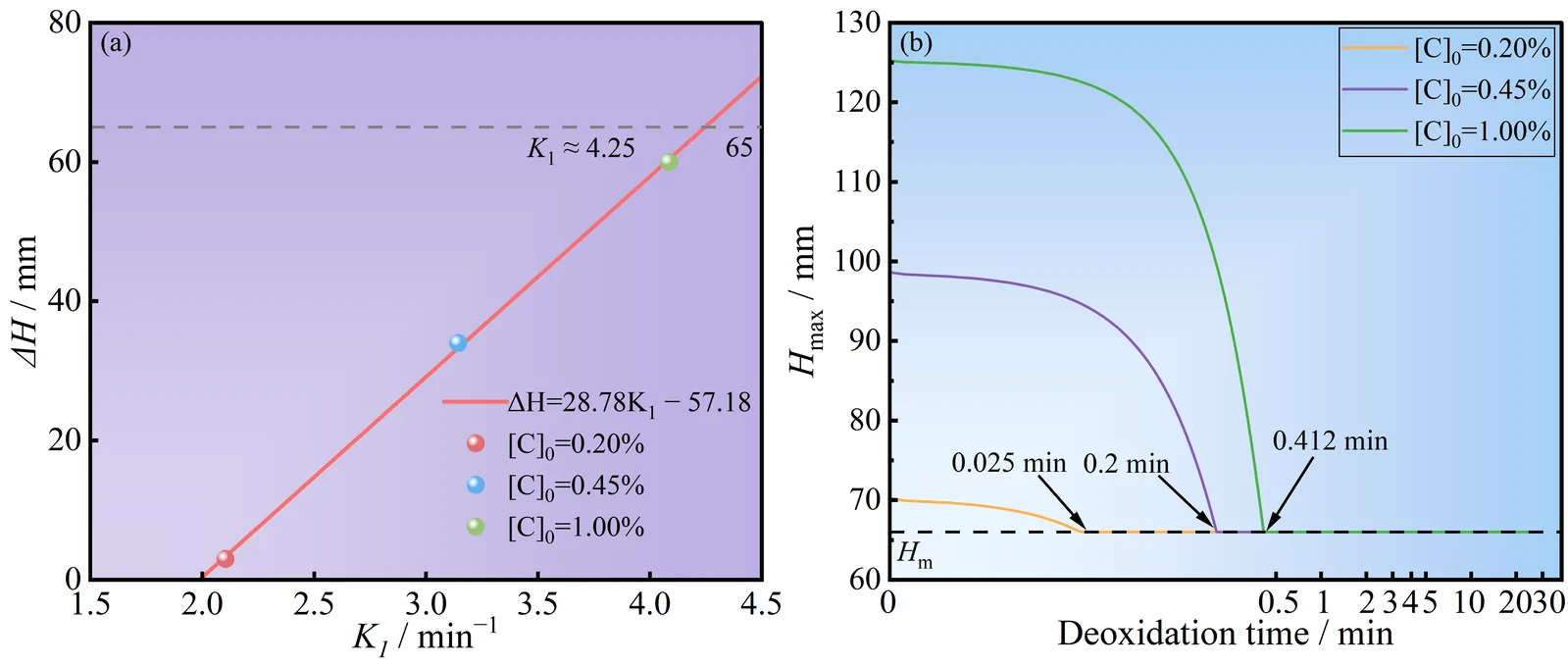
Relationship between internal deoxidation kinetics and melt-level behavior: (**a**) linear relationship between the experimentally determined maximum melt-level rise Δ*H* and the internal apparent deoxidation rate constant *K*_1_; (**b**) calculated temporal evolution of the maximum local melt-surface elevation *H*_max_ under different initial carbon contents.

The internal apparent deoxidation rate constant *K*_1_ characterizes the intensity of internal CO generation and therefore provides a kinetic indicator of splash risk. To relate the kinetic results to the observed melt-level response, the S1 rate constants obtained for the three experimental conditions were correlated with their respective measured maximum melt-level rises, as shown in Figure 9a, yielding:

(19) $$\Delta H=\max[0,28.78K_{1}-\;57.18]$$

where Δ*H* denotes the maximum melt-level rise in mm and *K*_1_ represents the internal deoxidation rate constant in min^−1^. The fitted relationship yields an *R*^2^ value exceeding 0.99 within the investigated experimental range. The critical splash-prevention internal apparent deoxidation rate constant *K*_1_ for the experimental crucible system is approximately 4.25 min^−1^.

To further characterize the temporal evolution of melt-level behavior, the maximum local melt-surface elevation, *H*_max_(t), was defined by Equation (20) as follows:

(20) $$H_{max}(t)=H_{m}+\Delta H(t)$$

where *H*_max_(t) denotes the maximum local melt-free surface elevation at time *t*, Δ*H*(t) denotes the corresponding maximum local melt-level rise, and *H*_m_ = 66 mm is the initial bath depth.

The calculation assumes that the local surface response follows the change in internal gas generation. The short delay caused by bubble nucleation, accumulation, and ascent is neglected.

As shown in Figure 9b, *H*_max_(t) has its maximum value at *t* = 0 and then decreases toward *H*_m_. At the start of deoxidation, the carbon–oxygen concentration product and *Π* are both at their maximum values. The thermodynamic driving force, effective reaction depth, and internal CO generation intensity are also greatest at this time. As deoxidation proceeds, CO generation intensity decreases and *H*_max_(t) falls rapidly.

A notable feature is that Δ*H* reaches zero before *Π* falls to unity. According to Equation (19), a detectable surface rise occurs only when *K*_1_ > 1.99 min^−1^. When *K*_1_ falls below this value, internal CO generation may remain thermodynamically feasible because *Π* > 1. However, the gas generation intensity is insufficient to sustain the gas holdup required for a detectable local surface rise. Therefore, Δ*H* reaches zero before *Π* decreases to unity.

### 4.4. Critical Splash-Prevention Criterion for Industrial Ladles

**Figure 10:**
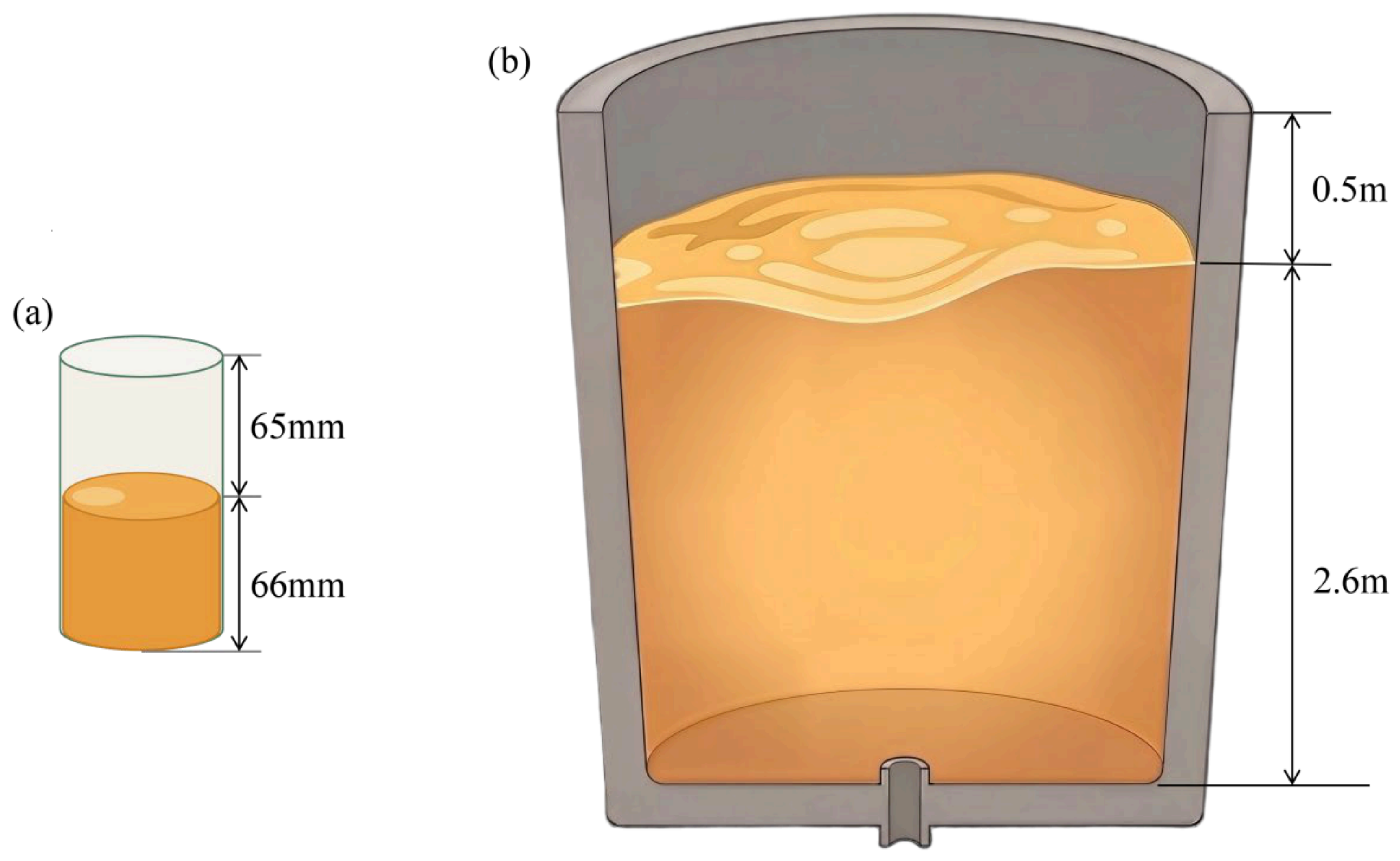
Bath geometry comparison: (**a**) laboratory crucible, (**b**) industrial ladle.

To evaluate the applicability of the proposed kinetic relationship under industrial conditions, an 85-ton steelmaking ladle was selected as the representative refining system. Following converter tapping, the bath depth *H*_m_ is approximately 2.6 m, while the freeboard height *H*_f_ between the melt surface and the ladle mouth is approximately 500 mm, as illustrated in Figure 10.

The absolute melt-level rise Δ*H* results from the increase in gas holdup *ε* produced by internal CO generation. Using the average gas holdup to describe the volumetric expansion of the bath, these quantities are related by the following:

(21) $$\Delta H\;={\;H}_{m}\frac{\varepsilon }{1\;-\;\varepsilon }$$

where *ε* is the average gas holdup in the bath.

Combining Equations (19) and (21) gives the following:

(22) $$\Delta H\;=\;H_{m}(0.4361K_{1}-\;0.8664)$$

Following macroscopic models of ladle metallurgy based on averaged gas variables [29,30], the present estimate uses the average gas holdup as the scale variable. Bubble expansion, drift, coalescence, and flow instability are not calculated separately in this estimate; their combined effect is represented by the average gas holdup. For a given *K*_1_, the industrial ladle and the laboratory crucible are assumed to have the same average gas holdup, and Equation (21) converts the relationship obtained from the crucible experiments to the ladle geometry. Because Δ*H* scales directly with bath depth *H*_m_, increasing *H*_m_ from 0.066 m in the laboratory crucible to 2.6 m in the industrial ladle amplifies the absolute melt-level rise by nearly fortyfold. However, the available freeboard remains relatively small compared with the bath depth. Consequently, the allowable internal deoxidation rate for splash prevention decreases from 4.25 to approximately 2.43 min^−1^.

The reaction-generated gas flow was evaluated from the internal oxygen removal during S1. The average internal oxygen removal rate is defined as follows:

(23) $$D_{[O]i}^{*}\;=\;\frac{{\Delta w}_{[O]i,S1}}{t_{S1}}$$

where $D_{[O]i}^{*}$ is the average internal oxygen removal rate during S1, in wt.%·s^−1^; Δ*w*_[O]i,S1_ is the cumulative decrease in oxygen content attributed to the internal pathway during S1, in wt.%; and *t*_S1_ is the duration of S1, equal to 30 s.

Because one mole of oxygen removed through the internal carbon–oxygen reaction produces one mole of CO, the molar CO formation rate is as follows:

(24) $${n˙}_{\mathrm{CO}}\;=\;\frac{\rho VD_{[O]i}^{*}}{100M_{O}}$$

where ${n˙}_{\mathrm{CO}}$ is the molar CO formation rate, in mol·s^−1^; *M*_O_ is the molar mass of oxygen, equal to 0.016 kg·mol^−1^; and the factor 100 converts wt.% to a mass fraction. For the industrial cases, *ρV* was set to the molten steel mass of 85,000 kg.

The equivalent CO volumetric flow rate at the bath temperature and at the pressure at half the bath depth is as follows:

(25) $$Q_{\mathrm{CO}}\;=\;\frac{{n˙}_{\mathrm{CO}}R_{g}T}{P_{0}\;+\;\frac{\rho gH_{m}}{2}}$$

where *Q*_CO_ is the equivalent CO volumetric flow rate, in m^3^·s^−1^, and *R*_g_ is the universal gas constant, equal to 8.314 J·mol^−1^·K^−1^.

The dimensionless gas flow rate is then calculated [29]:

(26) $$Q^{*}\;=\;\frac{Q_{\mathrm{CO}}}{\sqrt{g}{H_{m}}^{2.5}}$$

For the 0.45 wt.% C experiment, Q^*^ = 2.10 × 10^−2^. For the 85 t ladle, the Q^*^ values are 5.67 × 10^−2^ at 0.35 wt.% C and 0.0250 wt.% O and 4.66 × 10^−2^ at 0.40 wt.% C and 0.0210 wt.% O, respectively. The two industrial values are approximately 2.7 and 2.2 times the experimental value and remain within the same order of magnitude.

During production of medium-carbon steels such as 45 steel, the carbon content after converter tapping is typically adjusted to 0.35–0.40 wt.%. Accordingly, controlling the initial dissolved oxygen content *w*_[O]0_ at converter tapping provides an effective strategy for splash prevention. Inverse calculations using the dual-pathway kinetic model indicate that, to keep *K*_1_ strictly below 2.43 min^−1^ during ladle refining, the initial oxygen content must not exceed 0.0250 wt.% at the lower carbon limit of 0.35 wt.%. Because of the stronger supersaturation driving force, this limit should be reduced to 0.0210 wt.% at the upper carbon limit of 0.40 wt.%. To ensure splash-free refining of 45 steel over the entire composition range, the initial dissolved oxygen content at converter tapping should be kept below 0.0210 wt.%.

The proposed criteria are based on a single carbon addition during converter tapping. In industrial practice, staged recarburizer addition is expected to relax the constraints imposed by both ladle freeboard and the initial dissolved oxygen content. Future work will quantify the influence of staged recarburizer addition on the proposed splash-prevention limits.

## 5. Conclusions

Hot-state experiments and a dual-pathway kinetic model were used to investigate the relationship between carbon deoxidation kinetics and melt-level rise under atmospheric conditions. The main conclusions are as follows:

(1) The dual-pathway kinetic model separates atmospheric carbon deoxidation into free-surface mass transfer and internal CO generation at active refractory pores. A dimensionless carbon–oxygen supersaturation *Π* is introduced to characterize the thermodynamic driving force for CO bubble nucleation, while the effective reaction depth constrained by hydrostatic pressure describes the spatial extent of internal deoxidation.
(2) Using the calibrated internal deoxidation process coefficient k_a_ = 0.08 s^−1^ and the fitted equivalent oxygen contribution associated with crucible-melt interactions, the predicted oxygen evolution agrees well with the experimental measurements over the investigated carbon range of 0.20–1.00 wt.%, with a sum of squared relative errors of 0.126 and a mean relative error of 7.7%.
(3) Deoxidation proceeds through three distinct stages. Internal deoxidation initially dominates, with contribution fractions of 94.1–98.2% across the three carbon systems. As carbon–oxygen supersaturation decreases below the critical level, the nucleation zone retreats upward from the pool bottom and the effective reaction depth decreases. The dominant pathway then shifts from internal CO generation to free-surface mass transfer, and internal gas generation eventually ceases.
(4) Within the investigated experimental conditions, the internal apparent deoxidation rate constant *K*_1_ during the initial rapid stage shows an empirical relationship with the maximum melt-level rise Δ*H*. The critical splash-prevention rate for the experimental crucible is 4.25 min^−1^. By combining the experimental *K*_1_–Δ*H* relationship with the average gas holdup relation and the ladle geometry, the corresponding critical rate for the selected 85 t ladle is estimated to be approximately 2.43 min^−1^.

## Figures and Tables

**Table 1:** Geometric and thermophysical parameters of the crucible-molten steel system.

Parameter	Value	Unit
Radius of free surface (r)	0.025	m
Bath depth after melting (*H*_m_)	0.066	m
Freeboard height (*H*_f_)	0.065	m
Total internal height of crucible	0.131	m
Free surface area (*A*_s_)	1.96 × 10^−3^	m^2^
Volume of molten steel (*V*)	1.30 × 10^−4^	m^3^
Molten steel temperature (*T*)	1873	K
Ambient pressure (*P*_0_)	101,325	Pa
Density of molten steel (*ρ*)	7200	kg·m^−3^

**Table 2:** Proximate analysis of pulverized coal additive (wt.%).

Fixed Carbon	Ash	Volatile Matter	Moisture
92.36	5.46	1.62	0.34

## Data Availability

The original contributions presented in this study are included in the article. Further inquiries can be directed to the corresponding authors.

## Appendix A. Model Parameters

The symbols and parameters used in this study are summarized in Table A1. The table distinguishes experimentally specified parameters, calculated quantities, adopted model coefficients, and parameters determined by fitting in Section 4.1.

**Table A1:** Parameters used in the dual-pathway carbon deoxidation kinetic model.

Symbol	Value	Unit	Definition
T	1873	K	Thermodynamic temperature of the molten steel
*P*_0_	101,325	Pa	Ambient pressure
*ρ*	7200	kg·m^−3^	Density of the molten steel
*g*	9.81	m·s^−2^	Gravitational acceleration
*r*	0.025	m	Radius of the melt-free surface
*H*_m_	0.066	m	Initial bath depth
*A*_s_	1.96 × 10^−3^	m^2^	Area of the melt-free surface
*V*	1.30 × 10^−4^	m^3^	Volume of the molten steel
*K*	Calculated using Equation (2)	—	Carbon–oxygen equilibrium constant at 1873 K
*k*_s_	1.644 × 10^−4^ Ref. [24]	m·s^−1^	Oxygen mass-transfer coefficient at the gas–liquid interface
*k*_a_	0.08 Calibrated by trial and error	s^−1^	Calibrated process coefficient for the internal deoxidation pathway
*O*_cru_	1.21 × 10^−3^ Fitted parameter	wt.%	Fitted equivalent steady-state oxygen contribution associated with interactions between the crucible and the melt
*P*_CO,furnace_	0.044	atm	Fixed equivalent CO partial pressure used as the furnace gas boundary condition
*w*_[__C__]0_	0.20, 0.45, 1.00	wt.%	Initial carbon contents for the three calculation cases
*w*_[O]0_	0.0404, 0.0332, 0.0370	wt.%	Initial dissolved oxygen contents corresponding to the three calculation cases
*P*_CO_	Calculated using Equation (3)	atm	Equilibrium CO partial pressure corresponding to the instantaneous melt composition
*w*_[C]_	Time-dependent	wt.%	Instantaneous carbon mass fraction in the molten steel
*w*_[O]_	Time-dependent	wt.%	Instantaneous dissolved oxygen mass fraction in the molten steel
*P*_g_	Calculated using Equations (4) and (5)	Pa	CO gas pressure within a bubble
*h*	0–*H*_m_	m	Depth measured from the melt-free surface
*σ*	—	N·m^−1^	Surface tension of the molten steel used in the nucleation criterion
*r*_c_	Calculated using Equation (4)	m	Curvature radius of a CO bubble
*θ*	150	°	Contact angle between the molten steel and refractory pores
*R*	*R*_min_ < *R* < *R*_max_	m	Radius of an active refractory pore
*R*_max_	Calculated using Equation (6)	m	Upper pore-radius limit for retaining a gas nucleus
*R*_min_	Calculated using Equation (7)	m	Lower pore-radius limit for bubble growth and detachment
*H*	Time-dependent (Equation (8); 0–*H*_m_)	m	Effective depth within which internal deoxidation is thermodynamically feasible
*Π*	Time-dependent (Equation (9))	—	Global thermodynamic carbon–oxygen supersaturation index
*D*_[O]s_	Time-dependent (Equation (10)) Ref. [24]	wt.%·s^−1^	Free-surface deoxidation rate
$w_{[O],\mathrm{CO}}^{\mathrm{eq}}$	Calculated using Equation (11)	wt.%	CO equilibrium oxygen content
*w*_[O]e_	Calculated using Equation (12)	wt.%	Effective interfacial oxygen content
*D*_[O]i_	Time-dependent (Equation (13)) Refs. [14,25,26]	wt.%·s^−1^	Internal pore deoxidation rate
*w*_[O]_ *_h_* ^eq^	Depth-dependent (Equation (14))	wt.%	Static-pressure equilibrium oxygen content at depth h
*t*	0–1800	s	Calculation time
Δ*t*	0.1	s	Numerical time step
*t*_end_	1800	s	Total calculation duration
*t*_S1_	30	s	Duration of the rapid deoxidation stage S1
Δ*w*_[O]i,S1_	0.0251; 0.0136; 0.0111	wt.%	Cumulative internal oxygen removal during S1; values correspond to the 0.45 wt.% C experiment and the two 85 t ladle cases
$D_{[O]i}^{*}$	Calculated using Equation (23)	wt.%·s^−1^	Average internal oxygen removal rate during S1
*M*_O_	0.016	kg·mol^−1^	Molar mass of oxygen
*R*_g_	8.314	J·mol^−1^·K^−1^	Universal gas constant
${n˙}_{\mathrm{CO}}$	Calculated using Equation (24)	mol·s^−1^	Molar CO formation rate
*Q*_CO_	Calculated using Equation (25)	m^3^·s^−1^	Equivalent CO volumetric flow rate at bath temperature and pressure at half the bath depth
*Q* ^*^	Calculated using Equation (26) Ref. [29],	—	Dimensionless gas flow rate
